# Supplementary material for: Effect of Chitin Nanocrystals on Crystallization and Properties of Poly(lactic acid)-Based Nanocomposites
Source: Polymers (Basel). 2020 Mar 24;12(3):726. doi: 10.3390/polym12030726 (PMC7183044; doi:10.3390/polym12030726)
Supplement: Supplementary file 1 [file polymers-12-00726-s001.pdf]

polymers

Supplementary Materials:

Effect of chitin nanocrystals on crystallization and properties of poly(lactic acid)-based nanocomposites

Shikha Singh[1, 2], Mitul Patel[1], Daniel Schwendemann[1], Marta Zaccone[3], Shiyu Geng[1], Maria Lluisa Maspoch[2], and Kristiina Oksman[1, 4*]

[1] Division of Materials Science, Luleå University of Technology, SE-97 187 Luleå, Sweden
[2] Centre Català del Plàstic (CCP), Universitat Politècnica de Catalunya Barcelona Tech (EEBE-UPC), C/Colom, 114, Terrassa 08222, Spain
[3]Proplast, Via Roberto di Ferro 86, 15122 Alessandria (AL), Italy
[4]Mechanical & Industrial Engineering, University of Toronto, Toronto, M5S 3BS, Canada
[*]Correspondence: Kristiina.oksman@ltu.se; Tel: +46-920-493-371

Table S1. Optical properties of neat and isothermally crystallized PLA, PLA–TEC, and PLA–TEC–ChNC films at different temperatures and time periods

Materials	Transmittance (%)
PLA	92 (±0.25)
PLA-TEC	91 (±0.23)
PLA-TEC-ChNC	90 (±0.24)
PLA135-5	92 (±0.29)
PLA-TEC135-5	92 (±0.28)
PLA-TEC-ChNC135-5	90 (±0.29)
PLA135-15	91 (±0.26)
PLA-TEC135-15	91 (±0.47)
PLA-TEC-ChNC135-15	86 (±0.21)
PLA130-5	92 (±0.21)
PLA-TEC130-5	91 (±0.21)
PLA-TEC-ChNC130-5	89 (±0.29)
PLA130-15	93 (±0.61)
PLA-TEC130-15	92 (±0.58)
PLA-TEC-ChNC130-15	65 (±0.57)
PLA125-5	93 (±0.12)
PLA-TEC125-5	91 (±0.16)
PLA-TEC-ChNC125-5	85 (±0.18)
PLA125-15	91 (±0.20)
PLA-TEC125-15	90 (±0.46)
PLA-TEC-ChNC125-15	64 (±0.32)
PLA110	61 (±0.32)
PLA-TEC110	58 (±0.45)
PLA-TEC-ChNC110	69 (±0.44)

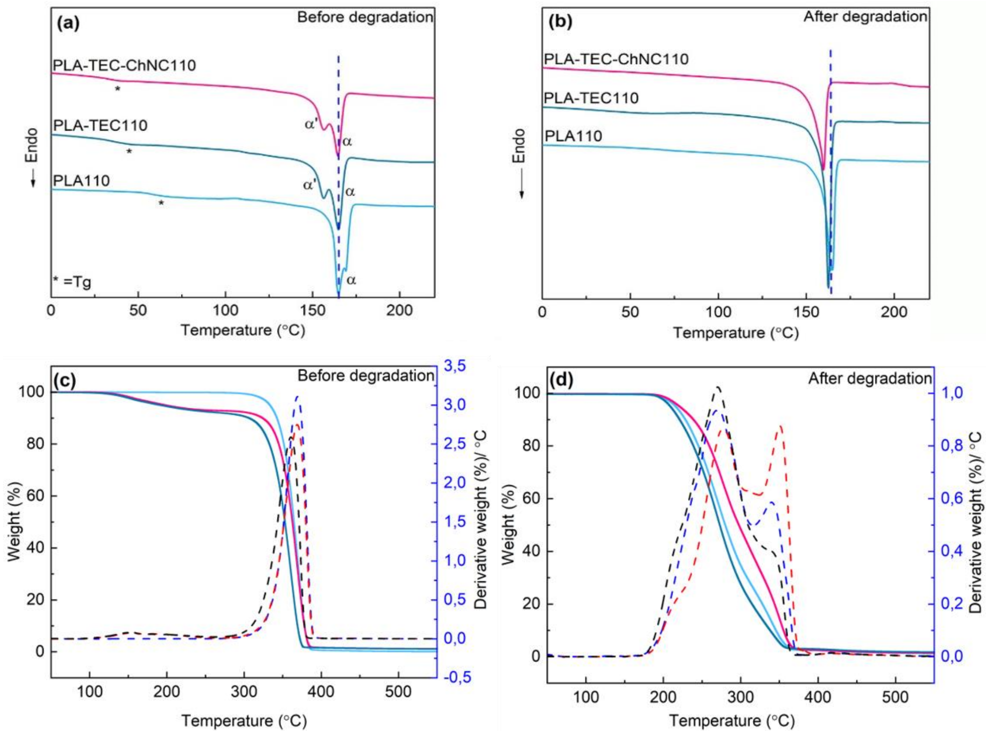

Figure S1. DSC thermograms of isothermally crystallized PLA110, PLA–TEC110, and PLA–TEC–ChNC110 films (a) before and (b) after hydrolytic degradation taken from first heating scans. TGA and DTG curves (dotted lines) of isothermally crystallized films (c) before and (d) after hydrolytic degradation
